# Prognostic Significance of Dynamic Free T3 Changes in Critically Ill Patients

**DOI:** 10.3390/clinpract16050087

**Published:** 2026-04-30

**Authors:** Yakup Özgüngör, Burak Emre Gilik, Emre Karagöz, Hicret Yeniay, Mensure Çakırgöz, Özlem Melis Korkmaz Özgüngör, İhsan Birol, Sıla Seven

**Affiliations:** 1 Department of General Intensive Care Unit, İzmir City Hospital, Izmir 35530, Turkey; dr.burakgilik@gmail.com (B.E.G.); dr.emrekaragoz@gmail.com (E.K.); drmensure@gmail.com (M.Ç.); silasevenn@gmail.com (S.S.); 2 Department of General Intensive Care, Balıkesir Atatürk City Hospital, Balıkesir 10185, Turkey; hicret.yeniay@yahoo.com; 3 Department of Occupational and Environmental Diseases, Dokuz Eylul University Faculty of Medicine, Izmir 35340, Turkey; omlskorkmazoz@gmail.com; 4 Department of Intensive Care, Suat Seren Chest Diseases and Thoracic Surgery Training and Research Hospital, Izmir 35110, Turkey; ihsanbirol@gmail.com

**Keywords:** non-thyroidal illness syndrome (NTIS), thyroid function tests, thyroid-stimulating hormone, 30-day mortality, APACHE, ICU, SOFA

## Abstract

**Background:** Non-thyroidal illness syndrome is frequent in critically ill patients, but the prognostic value of dynamic changes in thyroid function tests remains unclear. This study evaluated whether serial measurements of thyroid-stimulating hormone (TSH) and free triiodothyronine (FT3) provide additional predictive value for 30-day mortality beyond conventional severity scores in ICU patients. **Methods:** This single-center retrospective observational study included 74 adult patients treated for ≥72 h in a general ICU who had TSH and FT3 measured within 24 h of admission and repeated at 48–72 h. Patients aged 18 years or above admitted to the intensive care unit were included in the study. Demographic characteristics, comorbidities, APACHE II, SOFA, modified NUTRIC (mNUTRIC) scores, and routine laboratory data (including albumin, CRP, and lactate) were recorded. The primary outcome was 30-day mortality. Between-group comparisons were performed using t-tests, Mann–Whitney U, and Chi-square tests. Variables significant in univariate analyses were entered into binary logistic regression models, and predictive performance was assessed using receiver operating characteristic (ROC) curves and the Youden index. **Results:** The mean age was 68.7 ± 14.7 years, and 41.9% (*n* = 31) of the patients died within 30 days. Non-survivors had higher APACHE II, SOFA, and mNUTRIC scores and lower albumin, lymphocyte count, and second FT3 levels compared with survivors (all *p* ≤ 0.003). Baseline FT3 and TSH were not associated with mortality, whereas both the subsequent FT3 measurements and the ΔT3 (variance in former to latter FT3) were remarkably predictive. The latter FT3 < 1.63 pg/mL produced an AUC of 0.835 (sensitivity: 77%, specificity: 74%), and a ΔT3 log ratio threshold of −0.09 (≈20% early FT3 decline) produced an AUC of 0.835 (sensitivity: 71%, specificity: 81%). The APACHE II + ΔT3 (numeric) model showed the best discrimination (AUC: 0.921; sensitivity: 87.1%, specificity: 81.4%), outperforming APACHE II alone (AUC: 0.861). **Conclusions:** In critically ill adult patients, dynamic T3 kinetics—particularly premature decline in FT3 within the first 72 h—provide incremental prognostic value for 30-day mortality beyond APACHE II. Serial FT3 monitoring may help identify high-risk patients whose endocrine adaptation to critical illness is failing.

## 1. Introduction

Thyroid hormones play a fundamental role in sustaining numerous vital physiological processes, including regulation of energy metabolism, maintenance of hemodynamic stability, modulation of immune responses, and thermoregulation [1].

Thyroid function is regulated by the hypothalamic–pituitary–thyroid (HPT) axis. In this process, thyrotropin-releasing hormone (TRH) secreted by the hypothalamus stimulates the release of thyrotropin (TSH) from the anterior pituitary gland. TSH, in return, drives the thyroid gland to secrete its main hormones, thyroxine (T4) and triiodothyronine (T3). The prohormone T4 is converted into the biologically active hormone T3 in peripheral tissues [2,3,4].

In critical illness, numerous systemic alterations occur, including changes that affect the HPT axis as part of a broader neuroendocrine stress response involving increased catecholamines and glucocorticoids. Although these patients are clinically euthyroid, laboratory tests—particularly total T3 levels—often demonstrate values below the normal range. This condition, previously referred to as “euthyroid sick syndrome,” is now more commonly termed non-thyroidal illness syndrome (NTIS) [5]. The reduction in FT3 levels is primarily attributed to impaired peripheral conversion of T4 to T3, increased reverse T3 production, and alterations in deiodinase activity driven by inflammatory cytokines and stress-related hormonal changes [6].

NTIS has been extensively investigated in the literature, and its association with mortality has been well established [7]. The indication for thyroid hormone replacement therapy in patients with NTIS has also been examined, and it is widely accepted that such treatment does not offer clinical benefit [8].

Severity scoring systems such as the Acute Physiology and Chronic Health Evaluation II (APACHE II) and the Sequential Organ Failure Assessment (SOFA) scores are widely used to assess disease severity and predict mortality in critically ill patients [9,10]. APACHE II incorporates acute physiological parameters and chronic health status, while SOFA reflects the extent of organ dysfunction over time. Both scores have been consistently validated as reliable predictors of short-term mortality in intensive care settings.

Objective: We aimed to determine whether repeated measurements of TSH and T3 in intensive care patients could provide additional predictive value for 30-day mortality beyond established scoring systems such as APACHE II and SOFA.

## 2. Materials and Methods

This study was designed as a retrospective observational study. Ethical approval was obtained from the Non-Interventional Research Ethics Committee of İzmir City Hospital (Decision No: 2025/326; Date: 9 July 2025), and data collection was subsequently initiated. Patient consent is not required due to the retrospective design of the study. The study includes patients treated in the general intensive care unit of İzmir City Hospital between January 2024 and July 2025.

Inclusion criteria:

- Patients aged 18 years or above.
- Intensive care unit (ICU) stay of more than 72 h.
- Registration and calculation of APACHE-II and SOFA scores within the first 24 h of ICU admission.
- Availability of laboratory results for albumin, procalcitonin, C-reactive protein (CRP), white blood cell (WBC) count, and arterial blood gas analysis within the first 24 h of admission.
- Patients were included if they had at least one baseline measurement of TSH and FT3 on admission, as well as at least one follow-up measurement within 48–72 h obtained from the hospital database.

Exclusion criteria:

- Patients with a history of thyroidectomy, known autoimmune thyroid disease, or those receiving thyroid-associated medications (including antithyroid drugs or levothyroxine replacement therapy) were excluded from the study.
- Patients with TSH levels >10 mIU/L or <0.1 mIU/L, accompanied by abnormal FT4 levels, were excluded to rule out undiagnosed overt hypothyroidism or hyperthyroidism.
- Current treatment with amiodarone, corticosteroids (e.g., prednisolone), or dopamine.
- Hospital stay longer than 24 h prior to ICU admission.

TSH and free T3 values were recorded as two consecutive measurements throughout the intensive care follow-up. The initial measurements (TSH_1_ and FT3_1_) represent the baseline values obtained within the first 24 h of ICU admission, whereas the subsequent measurements (TSH_2_ and FT3_2_) refer to the follow-up values obtained between the 48 and 72 h window after admission. Due to the retrospective nature of the study, measurements were not obtained at fixed time points within this interval.

Given the fact that the absolute numerical differences between the two measurements were relatively small, both proportional and absolute changes were analyzed to better comprehend the kinetics of thyroid hormones. A logarithmic transformation was applied to convey the proportional changes more effectively. The change in TSH and free T3 was defined as follows:

ΔTSH = 〖log^ƒ()^〗_10 ((TSH_2)/(TSH_1))

ΔFT3 = 〖log^ƒ()^〗_10 ((FT3_2)/(FT3_1))

ΔFT3_numeric = FT3_2-FT3_1

The modified Nutrition Risk in Critically Ill (mNUTRIC) score was calculated retrospectively to assess the nutritional status of the patients.

Statistical analyses were performed using SPSS version 26 for Windows (IBM Corp., Armonk, NY, USA).

The data were analyzed in two categories: continuous and categorical variables. Continuous variables were expressed as means and standard deviations, while categorical variables were presented as frequencies and percentages. The normality of distribution for continuous variables was assessed using the Shapiro–Wilk test. For group comparisons, Student’s *t*-test was used for normally distributed variables, and the Mann–Whitney U test was applied for non-normally distributed variables. Categorical variables were analyzed using the Chi-square test.

Based on the univariate analyses, variables that were individually statistically significant (*p* < 0.05) were included in a binary logistic regression model. Odds ratios (OR) and 95% confidence intervals (CI) were calculated. Model calibration was assessed using the Hosmer–Lemeshow goodness-of-fit test. Variables that remained statistically significant in the multivariate model were further evaluated using receiver operating characteristic (ROC) curve analysis, and the optimal cut-off values were determined using the Youden index.

## 3. Results

Baseline characteristics of the study population are summarized in Table 1 and Table 2. Among the 74 patients, 50% were female (*n* = 37) and 50% were male (*n* = 37), with a mean age of 68.69 ± 14.67 years. The 30-day mortality rate was 41.9% (*n* = 31). Hypertension was the most prevalent comorbidity (60.8%, *n* = 45), followed by diabetes mellitus (52.7%, *n* = 39), coronary artery disease (20.3%, *n* = 15), chronic obstructive pulmonary disease (20.3%, *n* = 15), and chronic kidney disease (14.9%, *n* = 11).

Continuous clinical variables included APACHE-II, SOFA, and mNUTRIC scores; baseline and the 48 & 72 h window difference in TSH and FT3 levels; serum albumin, procalcitonin, creatinine, eGFR, C-reactive protein, white blood cell, neutrophil, lymphocyte, and platelet counts; serum lactate; the ICU length of total hospital stay, and time to death among non-survivors. Detailed distributions of these parameters are presented in Table 1.

According to the Mann–Whitney U test, the following variables were significantly associated with 30-day mortality: age (*p* = 0.006), latter TSH measurement (*p* = 0.018), APACHE-II score (*p* < 0.001), SOFA score (*p* = 0.004), and mNUTRIC score (*p* = 0.003), CRP (*p* = 0.021).

Based on the independent samples t-test, both the latter FT3 measurements (*p* < 0.001) and serum albumin levels (*p* = 0.001) showed eminent differences between survivors and non-survivors.

Detailed results for continuous variables are presented in Table 3, and categorical comparisons are summarized in Table 4.

In the subsequent phase, six distinct predictive models were developed by combining either the SOFA or APACHE-II scores with the latter FT3, ΔT3 (log ratio), ΔFT3 numeric or ΔTSH (log ratio) values. Each model was independently analyzed using binary logistic regression to identify independent predictors of 30-day mortality. Models in which all covariates demonstrated statistical significance (*p* < 0.05) were subsequently evaluated using receiver operating characteristic (ROC) curve analysis to determine the area under the curve (AUC) values.

In order to avoid the risk of multicollinearity and overfitting, the APACHE-II and SOFA scores were analyzed separately, and thyroid-associated parameters (TSH and T3 indices) were not entered into the same model. The mNUTRIC score was excluded from regression analyses since it incorporates elements derived from both the APACHE-II and SOFA systems. Given the observation of 31 mortality instances, all models were constructed with no more than three covariates, maintaining a ratio of at least 10 events per variable (EPV) to ensure statistical robustness. When serum albumin was incorporated into these models, it did not emerge as a prominent predictor in the binary logistic regression analysis, with a *p*-value of 0.056. The loss of significance of albumin in the multivariate model may indicate an overlap with FT3-related physiological pathways, particularly those linked to systemic inflammation and nutritional status. Albumin is a well-established marker of both nutritional reserve and inflammatory burden, and its dynamic changes have been associated with mortality in critically ill patients. In a previous study, albumin levels measured at 48 h were identified as independent predictors of 28-day mortality, emphasizing the importance of dynamic metabolic responses rather than baseline values [11].

Based on the ROC curve analysis, optimal cut-off points were determined using the Youden index. The ΔT3 (log ratio) produced an AUC of 0.835 (95% CI: 0.736–0.934), with the most appropriate cut-off identified as −0.09, corresponding to a sensitivity of 71% and specificity of 81%. This threshold presents approximately a 20% decline in FT3 levels prior to logarithmic transformation, suggesting that such a drop within 48 h of ICU admission is associated with a notably increased risk of mortality.

For the latter FT3 measurements, the AUC was 0.835 (95% CI: 0.744–0.926), and a value below 1.63 pg/mL served as the optimal mortality threshold (sensitivity: 77%, specificity: 74%).

In comparison, the ΔTSH (log ratio) demonstrated a modest discriminative capacity (AUC: 0.719, 95% CI: 0.602–0.837) with a cut-off of −0.17 (sensitivity: 45%, specificity: 77%).

When composite models were compared, the APACHE-II + Latter FT3 model exhibited the highest discriminative performance (AUC = 0.901, sensitivity: 87.1%, specificity: 72.1%), followed diligently by the APACHE-II + ΔT3 (log ratio) model (AUC = 0.850, sensitivity: 87.1%, specificity: 74.4%).

While the APACHE-II score alone maintained a high level of predictive accuracy (AUC = 0.861), the SOFA score demonstrated relatively limited discriminatory competence (AUC = 0.695).

Unanimously, these findings indicate that incorporating dynamic T3-related parameters—particularly ΔFT3_numeric and latter FT3 measurements—remarkably enhances the prognostic performance of established severity indices such as the APACHE-II score.

**Figure 1:**
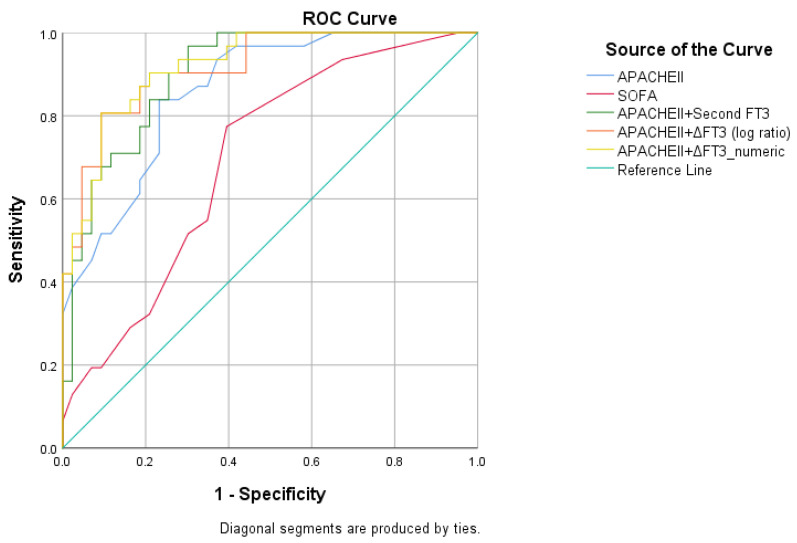
ROC analyses of different models. Receiver operating characteristic (ROC) curves comparing the predictive performance of different models for 30-day mortality. Models incorporating free triiodothyronine (FT3) and ΔT3 (log ratio) in combination with the APACHE-II score demonstrated superior discriminative ability compared with APACHE-II or SOFA scores alone.

The detailed logistic regression and ROC outcomes, including *p*-values, AUCs, Hosmer–Lemeshow goodness-of-fit results, and corresponding sensitivity and specificity values for each model, are summarized in Table 5, and the corresponding ROC curves are presented in Figure 1.

## 4. Discussion

In this study, we investigated the relationship between thyroid function tests—particularly early changes in free triiodothyronine (FT3) levels (T3 kinetics)—and short-term mortality among critically ill patients. Our findings demonstrated that it was not the baseline FT3 levels on ICU admission, but rather the following measurements obtained at the 48&72 h window and the variance in these two measurements (ΔT3), that served as strong predictors of mortality. Notably, the latter FT3 measurement achieved the highest discriminative competence both independently and when combined with the APACHE-II score, suggesting that dynamic assessment of thyroid function provides greater prognostic value for predicting clinical outcomes in critical illness than single-timepoint measurements [12]. Although the “APACHE II + ∆T3” model demonstrated the highest discriminative performance, its practical implementation in routine clinical settings may be limited due to the need for serial measurements and calculation of dynamic changes. Conversely, the “APACHE II + latter FT3” model offers a more straightforward and clinically applicable approach since it relies on a single follow-up measurement, which is easier to interpret at the bedside. Therefore, while dynamic models may provide superior statistical accuracy, more basic models based on single time-point measurements may be more feasible for day-to-day clinical use.

Non-thyroidal illness syndrome (NTIS) has long been recognized as a phenomenon reflecting the metabolic adaptation to severe illness. Numerous studies conducted in intensive care settings have reported an association between low FT3 levels and increased mortality [13,14]. Nonetheless, the majority of these studies were limited to single-timepoint measurements and did not account for the temporal trajectory of thyroid hormones. More recent prospective analyses have exhibited that a steady decline in FT3 levels during the course of illness is more closely associated with adverse outcomes, with measurements obtained around the fifth day providing the most accurate prediction of mortality [15,16]. Similarly, other research has indicated that NTIS alone may not serve as an independent predictor of mortality, but that dynamic monitoring of thyroid function offers valuable insight into the clinical course [17]. Another investigation assessed FT3 levels not as time-dependent variables but specifically on the first day of invasive mechanical ventilation—a point representing clinical deterioration—and revealed that FT3 measured at the time was tremendously associated with poor prognosis [18,19]. In accordance with this body of evidence, our study reinforces that a kinetic approach—capturing the rate and direction of change in hormone levels—provides greater prognostic information than a single baseline measurement.

Our findings also contribute to the long-standing debate on whether NTIS represents an adaptive energy-conservation response or a maladaptive endocrine failure during critical illness. In severe systemic stress, cytokines such as interleukin-6, tumor necrosis factor-α, and interferon-γ have been shown to suppress hypothalamic thyrotropin-releasing hormone (TRH) synthesis, while surging dopaminergic activity and elevated glucocorticoid levels plummet pituitary TSH secretion. Concurrently, decreased type 1 deiodinase and increased type 3 deiodinase activity in peripheral tissues limit the conversion of T4 to T3, leading to reduced plasma FT3 and accumulation of reverse T3 (rT3). Although this biochemical state may initially serve as an adaptive mechanism to reduce energy expenditure, prolonged persistence can impair cellular oxidative metabolism and immune function, thereby increasing mortality risk [18,20].

In our study, the additionally calculated ΔT3 log ratio displayed statistically significant results in both univariate and multivariate analyses, thereby improving the predictive performance of the evaluated models. However, while the APACHE II + Latter FT3 model achieved an AUC of 0.901, the APACHE II + ΔT3 (log ratio) model reached a slightly lower value of 0.850. This discrepancy may be attributed to the fact that logarithmic transformation—although beneficial for proportional standardization and variance reduction—may also limit the model’s predictive contribution by compressing interindividual variability [21]. In order to address this limitation, we analyzed models incorporating T3 kinetics both in proportional (logarithmic) and absolute (numeric) forms. The ΔT3 (log ratio) presents the relative proportional change between the two FT3 measurements, whereas the ΔT3 (numeric) parameter reflects the absolute difference between the second and the first FT3 levels. According to our results, the APACHE II + ΔT3 (numeric) model demonstrated an AUC of 0.921, with a sensitivity of 87.1% and a specificity of 81.4%. These findings indicate that the numeric ΔT3 model provides superior discriminative competence compared to the logarithmic ratio model (AUC = 0.850), highlighting the clinical relevance of evaluating absolute changes in T3 levels for mortality prediction in critically ill patients.

According to the ROC analysis, the optimal cut-off value for the latter free T3 (FT3) measurement, as determined by the Youden index, was 1.63 pg/mL, showing a sensitivity of 77.0% and a specificity of 74.0%. Similarly, the optimal threshold for ΔT3 (log ratio) was identified as –0.09, corresponding to a sensitivity of 71.0% and a specificity of 81.0%. This value presents approximately a 20% drop in FT3 levels within 48 h prior to logarithmic transformation, indicating that patients exhibiting a decline greater than this threshold had a notably increased risk of mortality. These findings are consistent with prior research reporting that a 15% reduction in FT3 levels between baseline and days 3–5 of ICU stay was remarkably associated with higher mortality [15]. Furthermore, a study conducted in Turkey identified cut-off values of 1.62 pg/mL for day 1 and 1.25 pg/mL for 48 h, respectively [16]. However, the mean age of that study population (75.8 ± 11.2 years) was notably higher, which may explain the greater decline in FT3 levels observed at 48 h. Nonetheless, determining the optimal FT3 cut-off point during the early ICU course will require large-scale, multicenter randomized controlled trials to validate these findings and refine clinical applicability.

As maintained by the ROC analysis, the optimal cut-off value for ΔT3 (numeric) determined by the Youden index was −0.19, corresponding to a sensitivity of 87.1% and a specificity of 62.8%. This finding indicates that patients whose FT3 levels failed to surge by at least 0.19 pg/mL—or remained stable or declined—within 48–72 h had a strikingly higher risk of 30-day mortality. The inability of FT3 to surge adequately during the early phase of critical illness likely reflects an insufficient adaptive thyroidal response and a limited systemic recovery capacity. Specifically, an early rise in FT3 may indicate restoration of cellular metabolism and neuroendocrine homeostasis, whereas persistently low or decreasing FT3 denotes progression to a maladaptive phase characterized by impaired metabolic flexibility and higher mortality risk. Instead of implying a direct causal relationship, this pattern may reflect the underlying severity of illness. Changes in FT3 levels likely represent a sensitive indicator of systemic stress and neuroendocrine adaptation. While a premature rise in FT3 may be associated with recovery of metabolic and physiological stability, persistently low or decreasing FT3 levels may indicate a more severe disease state. Thus, the ΔT3 (numeric) parameter should be interpreted primarily as a prognostic marker reflecting illness severity rather than a direct mediator of adverse outcomes [16].

Furthermore, the study cohort was derived from a general intensive care unit, comprising a broad and heterogeneous patient population rather than being restricted to a single specialty (medical, surgical, or disease-specific). This diversity reinforces the applicability of the results across a wide spectrum of critically ill patients.

Nevertheless, several limitations ought to be acknowledged. The single-center design and moderate sample size may limit the generalizability of our findings. In addition, the retrospective inclusion of only patients with available serial thyroid function measurements introduces a potential risk of selection and indication bias. In our ICU, thyroid function tests are routinely obtained at admission as part of the standard laboratory evaluation; however, repeat measurements are not performed according to a predefined protocol and instead arise from routine clinical practice. Therefore, follow-up testing may have been influenced by factors such as prolonged ICU stay, evolving physiological instability, or physician discretion. Consequently, patients with repeated measurements may represent a selected subgroup with greater disease severity or more complex clinical trajectories, which could partially influence the observed association between thyroid hormone kinetics and mortality. The absence of long-term follow-up beyond 30 days further restricts the evaluation of long-term prognostic value. The exclusion of patients receiving medications known to interfere with the hypothalamic–pituitary–thyroid axis, such as dopamine and corticosteroids, may further limit generalizability. Although this approach was necessary to reduce confounders, it may restrict the applicability of the results to the broader critically ill population.

Additionally, the 48–72 h window used for follow-up measurements represents another important limitation. In critically ill patients, endocrine and inflammatory responses can evolve rapidly over short time intervals, and measurements obtained at 48 h may not be physiologically equivalent to those obtained at 72 h. This temporal heterogeneity may introduce variability in the assessment of hormone kinetics and reduce the precision of dynamic modeling. However, due to the retrospective design, a broader time window was necessary to ensure an adequate sample size and to capture early endocrine adaptations during the acute phase of critical illness. The absence of long-term follow-up beyond 30 days further restricts the evaluation of long-term prognostic value.

The lack of reverse T3 (rT3) measurements and the absence of T4/FT4 ratio analysis limit a more comprehensive assessment of thyroid hormone metabolism. In addition, detailed endocrine profiling, including thyroid autoantibodies and imaging, was not available. Despite excluding patients with a known history of thyroid disease and overt dysfunction, the presence of undiagnosed or subclinical thyroid abnormalities cannot be entirely ruled out, particularly in elderly critically ill populations, and may have influenced the observed hormonal patterns.

Furthermore, although patients with known thyroid disease were excluded, the possibility of undiagnosed or subclinical thyroid dysfunction cannot be entirely eliminated. Subclinical thyroid abnormalities, including nodular disease and subtle molecular alterations, are highly prevalent in elderly populations and may not be clinically apparent at baseline. Importantly, emerging evidence suggests that even morphologically normal thyroid tissue may harbor significant molecular and signaling heterogeneity, which can influence endocrine behavior under stress conditions [22]. In addition, thyroid cells are highly sensitive to systemic stress, and their function is dynamically modulated by intracellular processes such as oxidative stress, endoplasmic reticulum stress, and mitochondrial signaling pathways [23].

These adaptive and maladaptive responses may alter hormone production, conversion, and peripheral regulation independently of overt thyroid disease. Therefore, the assumption of a completely normal baseline thyroid axis in critically ill patients may not be entirely valid. This underlying and often unrecognized endocrine heterogeneity may influence FT3 and TSH kinetics during critical illness and should be considered a potential confounder when interpreting the observed associations.

Another important consideration is the use of different mathematical representations of FT3 kinetics, including both logarithmic ratios and absolute numeric differences. This approach may raise concerns regarding potential overfitting or data-driven model selection. However, these transformations were not applied post hoc to maximize model performance. Logarithmic transformation was primarily used to normalize skewed distributions and to represent proportional changes in hormone levels, which is a standard approach in biomarker kinetics. In parallel, absolute numeric differences were analyzed to provide a more clinically intuitive interpretation of FT3 dynamics. Therefore, rather than reflecting data dredging, the use of dual representations aimed to provide complementary statistical and clinical perspectives on thyroid hormone kinetics.

Finally, the inability to quantitatively assess nutritional status and the full spectrum of inflammatory response may have contributed to the observed hormonal variability. Thereby, future multicenter studies with larger cohorts and comprehensive endocrine and metabolic profiling are warranted to validate and extend these findings.

## 5. Conclusions

In critically ill adults, dynamic T3 kinetics—particularly a premature decline in FT3 within the first 72 h—provide incremental prognostic value for 30-day mortality beyond established severity scores, especially APACHE II, and to a lesser extent SOFA. Serial FT3 monitoring may help identify high-risk patients with impaired endocrine adaptation to critical illness.

## Figures and Tables

**Table 1:** Descriptive Statistics of Clinical and Laboratory Parameters (*n* = 74).

Variable	Mean ± SD
Age (years)	68.69 ± 14.67
Procalcitonin (ng/mL)	1.68 ± 5.13
TSH (µIU/mL)	1.65 ± 1.36
Latter TSH (µIU/mL)	2.38 ± 4.21
Free T3 (pg/mL)	1.93 ± 0.70
Latter Free T3 (pg/mL)	1.80 ± 0.78
Creatinine (mg/dL)	1.30 ± 1.16
GFR (mL/min/1.73 m^2^)	70.97 ± 37.15
Albumin (g/L)	29.96 ± 6.19
CRP (mg/L)	107.74 ± 98.76
WBC (/µL)	12,223 ± 9426
Neutrophils (/µL)	9266 ± 6871
Lymphocytes (/µL)	1184 ± 1071
Platelet (10^3^/µL)	213.78 ± 120.70
APACHE II (score)	22.43 ± 9.38
SOFA (score)	4.99 ± 3.02
mNUTRIC (score)	3.96 ± 1.97
ICU Stay (days)	16.39 ± 13.95

Values are presented as means ± standard deviations.

**Table 2:** Frequency and Percentage Distribution of Categorical Variables (*n* = 74).

Variable	*n*	%
Female	37	50.0
Male	37	50.0
Diabetes Mellitus (DM)	39	52.7
Hypertension (HT)	45	60.8
Chronic Obstructive Pulmonary Disease (COPD)	15	20.3
Coronary Artery Disease (CAD)	15	20.3
Chronic Kidney Disease (CKD)	11	14.9
Vasopressor Requirement	9	12.2
Invasive Mechanical Ventilation (IMV)	15	20.3

Values are presented as numbers and percentages.

**Table 3:** Comparison of Continuous Variables Between Survivors and Non-Survivors, and Normality Assessment.

Variable	Survivors (Mean ± SD) (*n* = 43)	Non-Survivors (Mean ± SD) (*n* = 31)	*p*-Value
Age (years)	65.4 ± 14.3	73.2 ± 14.2	0.006
Procalcitonin (ng/mL)	1.18 ± 3.75	2.38 ± 6.59	0.153
TSH (µIU/mL)	1.56 ± 1.13	1.78 ± 1.63	0.861
Free T3 (pg/mL)	2.01 ± 0.71	1.80 ± 0.69	0.243
T4 (µg/dL)	1.31 ± 0.30	1.30 ± 0.26	0.991
Latter TSH (µIU/mL)	2.30 ± 3.75	2.49 ± 6.12	0.018
Latter Free T3 (pg/mL) **	2.15 ± 0.76	1.31 ± 0.50	0.001
ΔT3 (log ratio)	0.0321 ± 0.1434	−0.1389 ± 0.1299	<0.001
ΔTSH (log ratio)	0.1613 ± 0.4326	−0.1515 ± 0.7974	0.032
ΔFT3_numeric	−0.1405 ± 0.598	0.4952 ± 0.454	<0.001
Creatinine (mg/dL)	1.28 ± 1.13	1.32 ± 1.20	0.780
GFR (mL/min/1.73 m^2^)	71.6 ± 36.9	70.1 ± 38.1	0.934
Albumin (g/L) **	31.9 ± 5.9	27.3 ± 5.6	0.001
CRP (mg/L)	89.4 ± 96.9	133.2 ± 97.1	0.021
WBC (/µL)	10.099 ± 4.428	15.170 ± 13.165	0.021
Neutrophils (/µL)	7.934 ± 3.939	11.115 ± 9.332	0.153
Lymphocytes (/µL)	1.384 ± 1.109	904 ± 965	0.003
Platelet (×10^3^/µL)	229.3 ± 103.9	192.2 ± 139.7	0.055
APACHE-II (score)	17.5 ± 7.3	29.2 ± 7.5	<0.001
SOFA (score)	4.2 ± 2.7	6.0 ± 3.2	0.004
mNUTRIC (score)	3.4 ± 2.0	4.7 ± 1.6	0.003
ICU Stay (days)	19.9 ± 16.9	11.6 ± 5.6	0.015

** normally distributed values according to the Shapiro–Wilk normality test. Student’s *t*-test was used for normally distributed variables, and the Mann–Whitney U test was applied for non-normally distributed variables.

**Table 4:** Comparison of 30-day mortality according to categorical variables (Chi-Square Test).

Variable	Survivor *n* (%) (*n* = 43)	Non-Survivor *n* (%) (*n* = 31)	*p*-Value
Female	23 (53.5%)	14 (45.2%)	0.480
Male	20 (46.5%)	17 (54.8%)	0.480
Diabetes Mellitus (DM)	21 (48.8%)	14 (45.2%)	0.755
Hypertension (HT)	25 (58.1%)	20 (64.5%)	0.579
Chronic Obstructive Pulmonary Disease (COPD)	33 (76.7%)	26 (83.9%)	0.452
Coronary Artery Disease (CAD)	32 (74.4%)	27 (87.1%)	0.181
Chronic Kidney Disease (CKD)	35 (81.4%)	28 (90.3%)	0.287
Vasopressor use	14 (32.6%)	17 (54.8%)	<0.001
Invasive Mechanical Ventilation (IMV)	8 (18.6%)	23 (74.2%)	<0.001

Values are expressed as *n* (%), with percentages calculated within each group. Categorical variables were analyzed using the Chi-square test.

**Table 5:** Binary Logistic Regression Analysis for 30-Day Mortality.

Model	*p*-Value	AUC	Odds Ratio (OR)	95% Confidence Interval for OR	Hosmer–Lemeshow *p*	Sensitivity	Specificity
APACHE II	<0.001	0.861	1.215	1.114–1.324	0.491	81.0	76.0
SOFA	0.015	0.695	1.233	1.042–1.460	0.116	74.0	60.0
APACHE II + Latter FT3	0.007	0.901	0.157	0.041–0.606	0.951	87.1	72.1
SOFA + Latter FT3	0.757		0.099	0.029–0.339	0.836		
APACHE II + ΔTSH (log ratio)	0.507		0.623	0.230–1.687	0.263		
SOFA + ΔTSH (log ratio)	0.100		1.1	0.408–1.189	0.331		
APACHE II + ΔT3 (log ratio)	0.001	0.850	0.132	0.082–0.281	0.951	87.1	74.4
SOFA + ΔT3 (log ratio)	0.797		1.027	0.836–1.262	0.836		
APACHE II + ΔT3 (numeric)	0.02	0.921	1.11	1.11–1.414	0.829	87.1	81.4

## Data Availability

The data supporting the findings of this study are available from the corresponding author upon reasonable request. Due to ethical and institutional restrictions related to patient privacy and data protection regulations, the data are not publicly available.

## Abbreviations

The following abbreviations are used in this manuscript:

APACHE II	Acute Physiology and Chronic Health Evaluation II
AUC	Area Under the Curve
CAD	Coronary Artery Disease
CI	Confidence Interval
CKD	Chronic Kidney Disease
COPD	Chronic Obstructive Pulmonary Disease
CRP	C-Reactive Protein
DM	Diabetes Mellitus
eGFR	Estimated Glomerular Filtration Rate
EPV	Events Per Variable
FT3	Free Triiodothyronine
FT4	Free Thyroxine
GFR	Glomerular Filtration Rate
HPT axis	Hypothalamic–Pituitary–Thyroid Axis
HT	Hypertension
ICU	Intensive Care Unit
IMV	Invasive Mechanical Ventilation
mNUTRIC	Modified Nutrition Risk in Critically Ill Score
NTIS	Non-Thyroidal Illness Syndrome
OR	Odds Ratio
ROC	Receiver Operating Characteristic
rT3	Reverse Triiodothyronine
SD	Standard Deviation
SOFA	Sequential Organ Failure Assessment
T3	Triiodothyronine
T4	Thyroxine
TRH	Thyrotropin-Releasing Hormone
TSH	Thyroid-Stimulating Hormone
WBC	White Blood Cell
